# Publisher Correction: High salinity shelf water production rates in Terra Nova Bay, Ross Sea from high-resolution salinity observations

**DOI:** 10.1038/s41467-024-45425-6

**Published:** 2024-02-02

**Authors:** Una Kim Miller, Christopher J. Zappa, Arnold L. Gordon, Seung-Tae Yoon, Craig Stevens, Won Sang Lee

**Affiliations:** 1 Lamont-Doherty Earth Observatory of Columbia University, Palisades, NY USA; 2 Kyungpook National University, Daegu, South Korea; 3 National Institute of Water and Atmospheric Research, Wellington, New Zealand; 4 University of Auckland, Auckland, New Zealand; 5 Korea Polar Research Institute, Incheon, South Korea

**Keywords:** Physical oceanography, Physical oceanography

Correction to: *Nature Communications* 10.1038/s41467-023-43880-1, published online 16 January 2024

The original version of this Article contained an error in Eq. (8j) in the PDF version only, where parts of the equation were not displaying correctly and incorrectly read:

$${{{\rm{EoT}}}}=9\!\!:87\sin < {{{\rm{?thyc}}}}=12{{{\rm{?}}}} > 2{{{\rm{B}}}}7\!\!:53\cos < {{{\rm{?thyc}}}}=12{{{\rm{?}}}}\, 1\!\!:5\sin {{{\rm{B}}}}$$

The correct form of Equation (8j) is:

$${EoT}=9.87\sin 2B-7.53\cos B-1.5\sin B$$

This has been corrected in the PDF version of the Article.

