# Supplementary material for: High Salinity Shelf Water production rates in Terra Nova Bay, Ross Sea from high-resolution salinity observations
Source: Nat Commun. 2024 Jan 16;15:373. doi: 10.1038/s41467-023-43880-1 (PMC10791653; doi:10.1038/s41467-023-43880-1)
Supplement: Supplementary file 1 — Supplementary Information [file 41467_2023_43880_MOESM1_ESM.pdf]

Supplementary Information supporting the article:

High Salinity Shelf Water production rates in Terra Nova Bay, Ross Sea from high-resolution salinity observations

Una Kim Miller[1], Christopher J. Zappa[1], Arnold L. Gordon[1], Seung-Tae Yoon[2], Craig Stevens[3,4], Won Sang Lee[5]

[1]Lamont-Doherty Earth Observatory of Columbia University
[2]Kyungpook National University
[3]National Institute of Water and Atmospheric Research
[4]University of Auckland
[5]Korea Polar Research Institute

Corresponding Author: Una Kim Miller (ukm2103@columbia.edu)

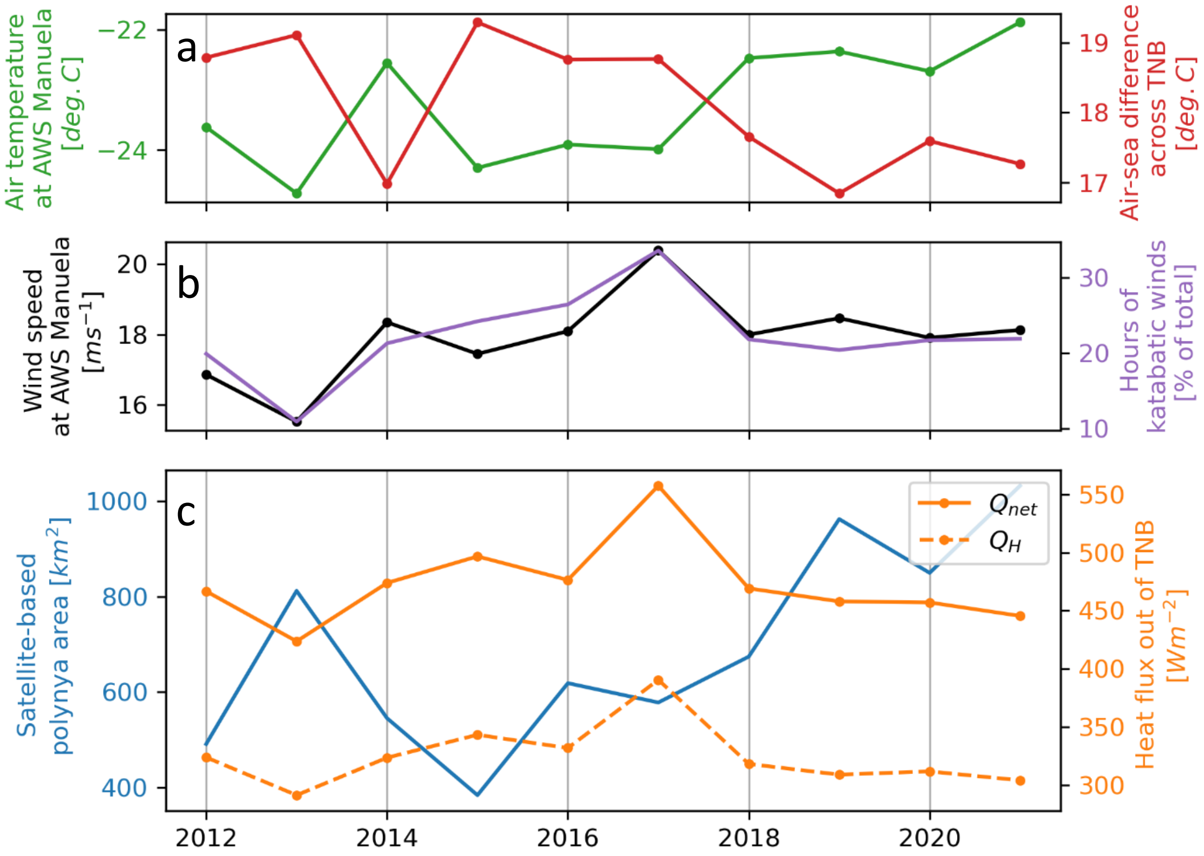

Supplemental Figure 1. Interannual timeseries of July – October average a) air temperatures at Automatic Weather Station (AWS) Manuela (green) and air-sea temperature difference calculated from European Center for Medium-Range Weather Forecasts (ECMWF) Reanalysis Version 5 (ERA5) and used to parameterize sensible heat fluxes (red), b) wind speeds at AWS Manuela (black) and cumulative number of hours of wind speeds $> 25 \mathrm{~m~s^{-1}}$ (katabatic winds) (purple), and c) polynya area calculated from satellite-derived sea ice concentration (SIC) maps (blue), parameterized net surface heat fluxes (orange solid line), and the sensible heat flux component (orange dashed line).

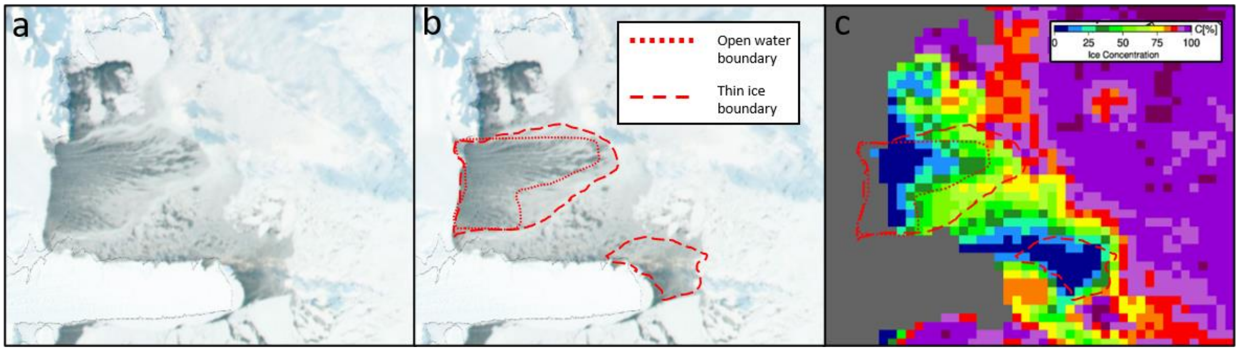

Supplemental Figure 2. Example comparison between a) visual imagery of the Terra Nova Bay Polynya and c) satellite-based, passive-microwave-derived sea ice concentrations (SIC) collected on the same day. Panel (b) shows the visually-identified open water area, corresponding to SIC of ~30%, outlined in the red dotted line, and the open water plus thin ice area, corresponding to SIC of ~60%, outlined in the dashed red line. The same outlines are recreated in Panel c.

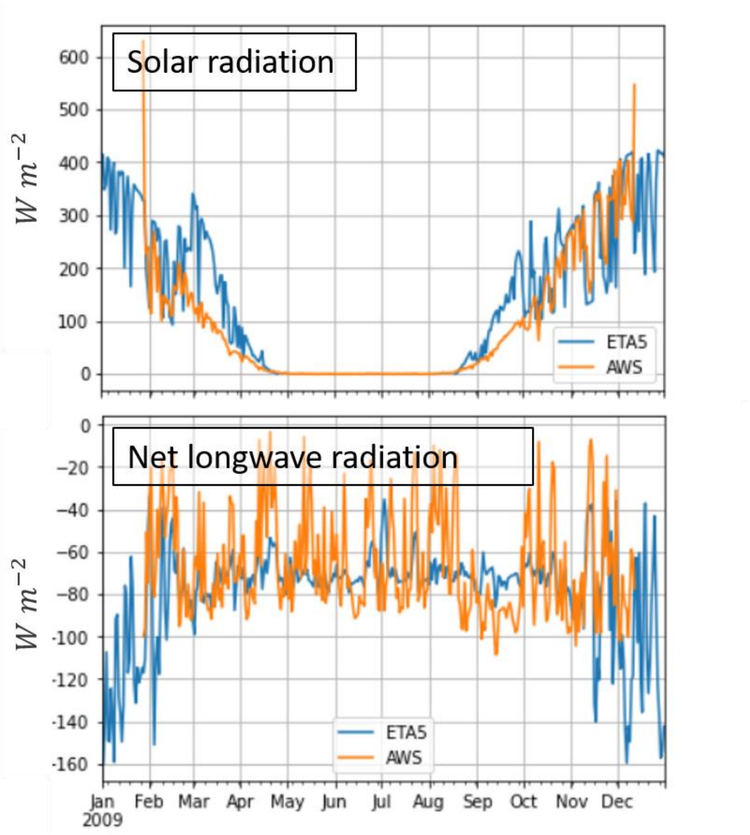

Supplemental Figure 3. A comparison of in-situ measurements of solar and net longwave radiation from Automatic Weather Station (AWS) Rita in 2009 (blue) with their parameterized values calculated over Terra Nova Bay using European Center for Medium-Range Weather Forecasts (ECMWF) Reanalysis Version 5 (ERA5) variables and AWS Rita wind speeds (orange).

Supplementary Table 1. Values of HSSW production rates, their major calculation componants (i.e., brine rejection rate dm/dt and polynya area), ice production rates, and katabatic wind statistics calculated in the week prior for each of the 27 identified HSSW production rate events.

	HSSW Production $[Sv]$	Ice Production Rate $[cm\ day^{-1}]$	dm/dt $[g\ m^{-2}s^{-1}]$	Polynya Area $[km^2]$	Average katabatic wind event duration $[hours]$	Average katabatic wind event strength $[m\ s^{-1}]$	Average katabatic wind event frequency $[week^{-1}]$
7/4/2017	2.1	75.9	0.2	429.7	7.9	27.3	7
7/7/2017	2.7	28.1	0.1	615.2	17.5	27.8	6
7/14/2017	2.4	87.1	0.2	478.5	14.6	29.7	5
7/14/2017	2.5	84.1	0.2	478.5	14.8	29.8	5
7/18/2017	2.4	57.7	0.2	752.0	33.3	30.6	3
7/30/2017	1.9	55.7	0.1	302.7	12.0	29.8	5
7/31/2017	0.8	20.9	0.1	312.5	9.9	29.1	7
7/31/2017*	0.9	20.9	0.1	371.1	9.9	29.1	7
8/8/2017	6.1	44.4	0.1	1601.6	7.0	27.0	11
8/14/2017	3.4	63.4	0.2	1162.1	12.5	28.4	4
8/20/2017	0.4	14.9	0.0	341.8	2.7	27.1	3
8/27/2017	3.4	36.2	0.1	556.6	4.9	27.1	8
8/28/2017	1.8	47.4	0.1	361.3	5.9	27.1	9
8/29/2017	2.1	38.8	0.1	273.4	9.5	27.3	6
9/2/2017	4.1	22.0	0.1	683.6	14.8	27.9	8
9/12/2017	1.0	69.0	0.2	175.8	9.9	29.0	7
9/13/2017	4.9	84.1	0.2	576.2	16.8	30.8	5
9/14/2017	5.5	75.6	0.2	966.8	21.4	31.8	5
9/18/2017	9.4	163.7	0.4	976.6	37.0	34.4	2
9/18/2017*	7.6	163.7	0.4	791.0	37.0	34.4	2
9/18/2017	7.3	221.7	0.6	449.2	37.0	34.6	2
9/22/2017	3.2	82.9	0.2	341.8	17.4	29.5	5
9/26/2017	2.5	17.8	0.0	166.0	10.9	27.9	7
9/30/2017	4.2	73.2	0.2	312.5	4.3	26.1	4
10/12/2017	4.5	72.9	0.2	263.7	8.0	26.4	1
10/18/2017	4.6	34.2	0.1	507.8	14.5	29.5	2
10/30/2017	10.0	62.2	0.2	2246.1	4.5	28.0	2
Average:	3.8	67.3	0.2	610.9	14.7	29.2	5.1
